# Analysis of the cost‐effectiveness of proton beam therapy for unresectable pancreatic cancer in Japan

**DOI:** 10.1002/cam4.6611

**Published:** 2023-10-05

**Authors:** Yuichi Hiroshima, Masahide Kondo, Takuya Sawada, Shu‐ling Hoshi, Reiko Okubo, Takashi Iizumi, Haruko Numajiri, Toshiyuki Okumura, Hideyuki Sakurai

**Affiliations:** ^1^ Department of Radiation Oncology & Proton Medical Research Center, Faculty of Medicine University of Tsukuba Tsukuba Ibaraki Japan; ^2^ QST hospital, National Institutes for Quantum and Radiological Sciences and Technology Chiba Chiba Japan; ^3^ Department of Radiation Oncology, Ibaraki Prefectural Central Hospital Kasama Ibaraki Japan; ^4^ Department of Health Care Policy and Health Economics, Faculty of Medicine University of Tsukuba Tsukuba Ibaraki Japan; ^5^ Department of Clinical Laboratory Medicine University of Tsukuba Hospital Tsukuba Ibaraki Japan; ^6^ Department of Nephrology, Faculty of Medicine University of Tsukuba Tsukuba Ibaraki Japan

**Keywords:** chemoradiotherapy, cost‐effectiveness, incremental cost‐effectiveness ratio, locally advanced pancreatic cancer, particle radiotherapy, proton beam therapy, quality‐adjusted life year, radiotherapy

## Abstract

**Background:**

Proton beam therapy (PBT) has recently been included in Japan's social health insurance benefits package. This study aimed to determine the cost‐effectiveness of PBT for unresectable, locally advanced pancreatic cancer (LAPC) as a replacement for conventional photon radiotherapy (RT).

**Methods:**

We estimated the incremental cost‐effectiveness ratio (ICER) of PBT as a replacement for three‐dimensional conformal RT (3DCRT), a conventional photon RT, using clinical evidence in the literature and expense complemented by expert opinions. We used a decision tree and an economic and Markov model to illustrate the disease courses followed by LAPC patients. Effectiveness was estimated as quality‐adjusted life years (QALY) using utility weights for the health state. Social insurance fees were calculated as the costs. The stability of the ICER against the assumptions made was appraised using sensitivity analyses.

**Results:**

The effectiveness of PBT and 3DCRT was 1.67610615 and 0.97181271 QALY, respectively. The ICER was estimated to be ¥5,376,915 (US$46,756) per QALY. According to the suggested threshold for anti‐cancer therapy from the Japanese authority of ¥7,500,000 (US$65,217) per QALY gain, such a replacement would be considered cost‐effective. The one‐way and probabilistic sensitivity analyses demonstrated stability of the base‐case ICER.

**Conclusion:**

PBT, as a replacement for conventional photon radiotherapy, is cost‐effective and justifiable as an efficient use of finite healthcare resources. Making it a standard treatment option and available to every patient in Japan is socially acceptable from the perspective of health economics.

## 1 INTRODUCTION

Pancreatic cancer is one of the most common causes of cancer death and has a poor prognosis. The number of cases and deaths annually continues to increase in Japan and worldwide.^1^, ^2^, ^3^ It is widely known that the prognosis of unresectable, locally advanced pancreatic cancer (LAPC) is extremely poor, and there is a great demand to improve the current treatment methods. The standard treatment for LAPC is chemotherapy or chemoradiotherapy.^4^, ^5^ Despite advances in cytotoxic anti‐cancer drugs, chemotherapy is lacking prominent progress, with no effective immune checkpoint inhibitors.^6^, ^7^, ^8^, ^9^, ^10^ On the contrary, chemoradiotherapy includes newly developed technologies such as intensity modulated radiotherapy (IMRT) and stereotactic body radiotherapy (SBRT); these techniques have enabled increased radiation dose exposure to tumors; however, their results have not been remarkable.^11^, ^12^, ^13^, ^14^, ^15^, ^16^

Recently, an increasing number of reports have used particle beam therapy to treat LAPC.^17^, ^18^, ^19^, ^20^, ^21^ Particle beam therapy can minimize the effect on surrounding organs while concentrating irradiation on the lesion. Therefore, it is possible to increase the radiation dose to the tumor and reduce adverse effects on the surrounding organs; good treatment results have been reported.^20^, ^21^, ^22^, ^23^, ^24^, ^25^ However, particle beam therapy is more expensive in terms of equipment and power, including the accelerator and beam transport system, in comparison with a standard photon radiotherapy. Attention is being paid to its cost‐effectiveness and whether it should be considered as one of the standard treatment options available to every patient.

In April 2022, Japan expanded their social health insurance benefit package to include proton beam therapy (PBT), which is a type of particle beam therapy. LAPC was included as a new target disease for PBT along with three other cancers (unresectable hepatocellular carcinoma with a length greater than 4 cm, unresectable intrahepatic cholangiocarcinoma, and unresectable postoperative locally recurrent rectal carcinoma), in addition to four existing target diseases (pediatric localized solid malignant tumor, unresectable bone and soft tissue tumor, head and neck tumors other than squamous cell carcinoma, and prostate cancer without metastasis) without any appraisal of its cost‐effectiveness. Therefore, we decided to evaluate the cost‐effectiveness of PBT for LAPC, as a replacement for conventional photon radiotherapy (RT).

The purpose of this study was to determine if such a replacement was socially and economically acceptable in Japan, thereby, providing insightful information to other health systems developing PBT.

## 2 METHODS

### 2.1 Literature review

We conducted an analysis on the cost‐effectiveness of introducing PBT into the current regimen of LAPC treatment in Japan. We performed a literature review to determine the best available clinical evidence. We accessed the PubMed database and Igaku Chuo Zasshi (Japana Centra Revuo Medicina), a Japanese medical literature database, with combinations of relevant terms such as locally advanced pancreatic cancer, radiotherapy, and proton beam therapy.

### 2.2 Decision tree and Markov model

**FIGURE 1:**
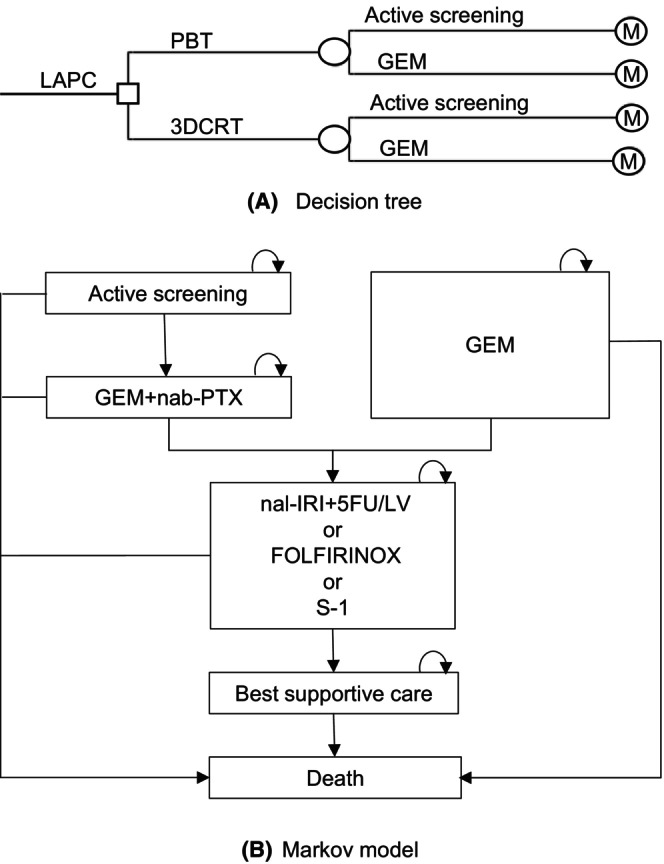
Models used to estimate the cost‐effectiveness of proton beam therapy. After receiving proton beam therapy (PBT) or three‐dimensional conformal radiotherapy (3DCRT), 80% patients underwent active screening and gemcitabine (GEM) regimen. Upon progression of disease, they moved into a Markov model. Patients under active screening moved to treatment with GEM + nab‐Paclitaxel (nab‐PTX) regimen upon progression. After receiving either GEM or GEM + nab‐PTX regimen, patients proceeded to treatment with one of any three regimens upon progression: nanoliposomal formulation of irinotecan, 5‐fluorouracil and leucovorin (nal‐IRI + 5FU/LV), 5‐fluorouracil/leucovorin combined with irinotecan and oxaliplatin (FOLFIRINOX), or S‐1 regimen. The proportions of receiving nal‐IRI + 5FU/LV, FOLFIRINOX, or S‐1 regimen were at 50%, 25%, and 25%, respectively. Upon further progression, they proceeded to best supportive care. Transitions to death from any of the above health states were assumed.

We compared PBT with three‐dimensional conformal radiotherapy (3DCRT), a conventional photon RT. We used a decision tree with a Markov model to estimate the incremental cost‐effectiveness ratios (ICER) of PBT replacing 3DCRT (Figure 1). These models were used to illustrate the disease courses of LAPC patients and were built according to expert opinions formed by three oncologists on LACP care at the University of Tsukuba Hospital.

In the decision tree model, we assumed that some patients underwent active screening after receiving PBT or 3DCRT, while others underwent a gemcitabine (GEM) regimen. In the Markov model, patients undergoing active screening were treated with the GEM + nab‐paclitaxel (nab‐PTX) regimen upon disease progression. Patients treated with the GEM + nab‐PTX regimen or GEM regimen were treated with one of the following three regimens upon progression: a nanoliposomal formulation of irinotecan, 5‐fluorouracil, and leucovorin (nal‐IRI + 5FU/LV); 5‐fluorouracil/leucovorin combined with irinotecan and oxaliplatin (FOLFIRINOX); or a S‐1 regimen. Upon further disease progression, they were moved to best supportive care (BSC). Some patients died during the course of treatment.

Outcomes in terms of quality‐adjusted life years (QALYs) were calculated by assigning probabilities and utility weights to these models. The Markov cycle for each stage was set at 1 month, with the model programmed to cease when the cohort's stage reached the 60th month. During the last cycle, all surviving patients were assumed to have died. The variable assumptions are summarized in Table 1.

**TABLE 1:** Model assumptions.

Variable	Value	Reference
Decision tree		
Probability from PBT or 3DCRT to Active screening	0.8	Expert opinion
Probability from PBT or 3DCRT to GEM	0.2	Expert opinion
Markov model		
Transition probability from PBT‐Active screening to GEM+nab‐PTX (MST)	0.08201 (8.1 months)	20
Transition probability from 3DCRT‐Active screening to GEM+nab‐PTX (MST)	0.12479 (5.2 months)	27
Transition probability from GEM+nab‐PTX to nal‐IRI + 5FU/LV, FOLFIRINOX or S‐1 (MST)	0.09178 (7.2 months)	28
Transition probability from PBT‐GEM to nal‐IRI + 5FU/LV, FOLFIRINOX or S‐1 (MST)	0.05005 (13.5 months)	20
Transition probability from 3DCRT‐GEM to nal‐IRI + 5FU/LV, FOLFIRINOX or S‐1 (MST)	0.07658 (8.7 months)	27
Share among nal‐IRI + 5FU/LV, FOLFIRINOX, or S‐1	0.5, 0.25, 0.25	Expert opinion
Transition probability from nal‐IRI + 5FU/LV to BSC (MST)	0.20036 (3.1 months)	9
Transition probability from FOLFIRINOX to BSC (MST)	0.16674 (3.8 months)	29
Transition probability from S‐1 to BSC (MST)	0.29289 (2.0 months)	30
Transition probability to Death with PBT (MST)	0.02671 (25.6 months)	20
Transition probability to Death with 3DCRT (MST)	0.05613 (12.0 months)	26
Utility weight		
LAPC	0.820	31
BSC	0.584	32
Costs		
PBT (Reimbursable fee for beam therapy + GEM and accompanying care)	¥2,430,261 (¥2,375,000 + ¥55,261)	Social insurance fee
3DCRT (Reimbursable fee radiation therapy + GEM and accompanying care)	¥749,411 (¥694,150 + ¥55,261)	Social insurance fee
Active screening (monthly)	¥17,115	Social insurance fee
GEM+nab‐PTX (monthly)	¥555,523	Social insurance fee
GEM (monthly)	¥74,827	Social insurance fee
nal‐IRI + 5FU/LV (monthly)	¥869,758	Social insurance fee
FOLFIRINOX (monthly)	¥229,128	Social insurance fee
S‐1 (monthly)	¥68,957	Social insurance fee
CV port placement	¥169,000	Social insurance fee
Monthly cost of BSC (monthly)	¥156,072	33
Terminal care for death during treatment	¥1,483,700	34
Terminal care for death from BSC	¥1,316,600	34
Discounting		
Discount rate	3%	

Abbreviations: 3DCRT, three‐dimensional conformal radiotherapy; BSC, best supportive care; FOLFIRINOX, 5‐fluorouracil/leucovorin combined with irinotecan and oxaliplatin; GEM, gemcitabine; LAPC, locally advanced pancreatic cancer; MST, median survival time; nab‐PTX, nab‐Paclitaxel; nal‐IRI + 5FU/LV, nanoliposomal formulation of irinotecan, 5‐fluorouracil and leucovorin; PBT, proton beam therapy.

### 2.3 Outcomes

The effectiveness of prolonging life with PBT was modeled as the difference in transition probability to death based on clinical evidence reported by Hiroshima et al.^20^ We assumed constant transition probabilities calculated based on the median survival times (MST), which were 25.7 and 12.0 months for PBT and 3DCRT, respectively.^20^, ^26^ The reason for selecting these reports from among many is that although there are reports of prospective clinical trials of 3DCRT for LAPC, no such studies of PBT have been reported to best of our knowledge, and only retrospective observational studies have been reported. Therefore, these reports were selected as a result of adopting reports of both observational studies and those with a large number of cases. Different transition probabilities were also calculated based on the assumed MST from active screening to GEM + nab‐PTX and from GEM to nal‐IRI + 5FU/LV, FOLFIRINOX, or S‐1 according to the modality of radiotherapy. The MST for the former was 8.1 months for PBT and 5.2 months for 3DCRT, while those for the latter were 13.5 months and 8.7 months, respectively.^20^, ^26^, ^27^ Regardless of PBT or 3DCRT intervention, the common probabilities of active screening and GEM in the decision model were assumed to be 0.8 and 0.2, respectively, after consultation with chemotherapy experts affiliated with our institution. Common transition probabilities were calculated based on the assumed MST from GEM + nab‐PTX to nal‐IRI + 5FU/LV, FOLFIRINOX, or S‐1, which was 7.2 months.^28^ The shares among the three regimens were assumed to be 0.5, 0.25, and 0.25, respectively, after consultation with experts. Common MSTs to BSC for the three regimens were assumed to be 3.1, 3.8, and 2.0 months, respectively.^9^, ^29^, ^30^

For the QALYs, utility weights for LAPC and BSC were assumed to be 0.820 and 0.584, respectively.^31^, ^32^

### 2.4 Costing

Cost items were identified using the decision tree and Markov models. Direct medical costs borne by social insurers and patients were considered, as these two entities constitute the major payers of healthcare in Japan's social health insurance system. The costs of PBT were set at a reimbursable fee of ¥2,375,000 (US$20,652, US$1 = ¥115), with GEM and accompanying care such as periodic outpatient examinations at ¥55,261 (US$480). The 3DCRT costed ¥694,150 (US$6036), with GEM and accompanying care valued at ¥55,261 (US$480). Accompanying care was modeled according to expert opinion, and reimbursable fees were summed according to the social insurance fee schedule, which were confirmed by an insurance claim officer at the hospital. The monthly costs of active screening and chemotherapy were set similar to the cost of accompanying care. The monthly costs of active screening, GEM+nab‐PTX, GEM, nal‐IRI + 5FU/LV, FOLFIRINOX, and S‐1 were set at ¥17,115 (US$148), ¥555,523 (US$4830), ¥74,827 (US$650), ¥869,758 (US$7563), ¥229,128 (US$1992), and ¥68,957 (US$599), respectively. The cost of central venous (CV) port placement was set at ¥169,000 (US$1469), which was added to the nal‐IRI + 5FU/LV and FOLFIRINOX regimen for patients. Thus far, cancer treatment has been offered as an outpatient service. The monthly cost of BSC, ¥156,072 (US$1357), was cited from the study by Ashino et al.^33^ who reported on home medical care costs for terminally staged cancer patients. The costs of palliative care, ¥1,483,700 (US$12,901), and death from BSC, ¥1,316,600 (US$11,448), were cited from the study by Hashimoto et al.^34^ who reported on hospitalization costs at the time of death. The former included costs of treatment for complications during chemoradiotherapy and chemotherapy, while the latter included the costs of palliative care after BSC.

### 2.5 ICERs estimation

ICERs, which were calculated as (total cost under PBT—total cost under 3DCRT) divided by (QALY gained under PBT—QALY gained under 3DCRT), were used to investigate whether replacing 3DCRT with PBT yielded sufficient improvement in outcomes and justified the increase in costs. The outcomes and costs were discounted at an annual rate of 3%. The model was parameterized using the TreeAge Pro software (version 2020; TreeAge, Inc.). The Central Social Insurance Medical Council in Japan set ¥7,500,000 (US$65,217) per QALY gain as a threshold for evaluating the cost‐effectiveness of anti‐cancer interventions from payers' perspective; we referred to this value for judging the cost‐effectiveness.^35^

### 2.6 Sensitivity analyses

To appraise the stability of ICER with assumptions made in our economic model and to explore the impact of each variable relative to each other, we performed one‐way and probabilistic sensitivity analyses (PSA), that is, 1000 Monte Carlo simulations. The lower and upper limits were ± 50% of the base‐case value for cost items and ± 20% for both the probabilities and utility weights. The discount rate varied from 0% to 5%. A triangular distribution was used for each variable in the PSA.

We also conducted a scenario sensitivity analysis to determine the different reimbursable fee levels for PBT. In the social insurance fee schedule, the two PBT fee levels were set for rare and non‐rare cancers. Currently, among the eight target diseases, only prostate cancer is categorized as non‐rare cancer, for which the fee is lower. Because LAPC was categorized as a rare cancer, we set our base‐case value at ¥2,375,000. In this scenario sensitivity analysis, we assumed the fee level for a non‐rare cancer treatment to be ¥1,600,000.

## 3 RESULTS

Table 2 shows the results of the cost‐effectiveness analysis of PBT replacing 3DCRT. The effectiveness of PBT and 3DCRT were 1.67610615 and 0.97181271 QALY, respectively, which does not contradict the difference in MST and resulted in a positive incremental effect of 0.70429344 QALY. The ICER was estimated at ¥5,376,915 per QALY. According to the suggested willingness‐to‐pay for one QALY gain, ¥7,500,000/QALY, PBT replacing 3DCRT would be considered cost‐effective.

**TABLE 2:** Cost‐effectiveness of PBT and 3DCRT.

	Cost (¥)	Incremental cost (¥)	Effectiveness (QALY)	Incremental effectiveness (QALY)	ICER (¥/QALY)
Proton beam therapy (PBT)	10,876,921	3,786,926	1.67610615	0.70429344	5,376,915
Conventional photon radiotherapy (3DCRT)	7,089,995		0.97181271		

Abbreviations: 3DCRT, three‐dimensional conformal radiotherapy; ICER, Incremental cost‐effectiveness ratio; QALY, Quality‐adjusted life year.

**FIGURE 2:**
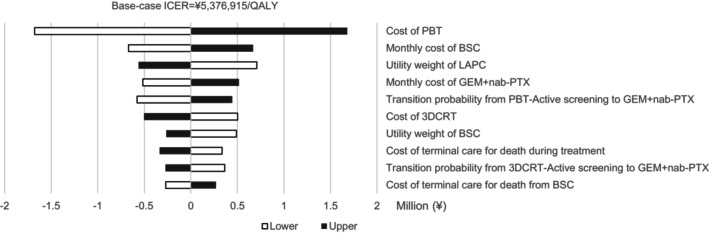
Results of one‐way sensitivity analysis. A total of 10 variables with large changes in incremental cost‐effectiveness ratio (ICER) from the base‐case value are shown. The ICER ranged from ¥3,697,680/quality‐adjusted life year (QALY) to ¥7,056,151/QALY, based on change in the cost of proton beam therapy (PBT). The upper limit of the cost of PBT yielded an ICER less than the threshold of ¥7,500,000/QALY.

Figure 2 shows 10 variables with large changes in ICER from the base‐case value from the one‐way sensitivity analysis. The ICER was found to be most sensitive to the cost of PBT, ranging from ¥3,697,680/QALY to ¥7,056,151/QALY. The upper limit of the cost of PBT yielded an ICER below the threshold of ¥7,500,000/QALY.^35^

**FIGURE 3:**
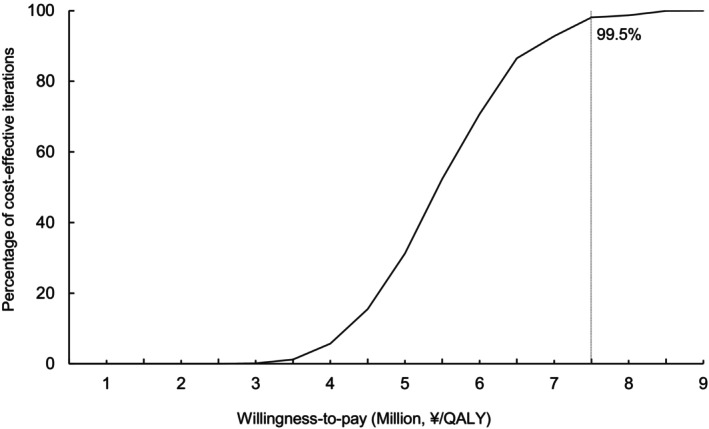
Results of probabilistic sensitivity analyses: cost‐effectiveness acceptability curve (CEAC). Among 1,000 incremental cost‐effectiveness ratios (ICERs) produced by the Monte Carlo simulation, the probability that the ICER is under ¥7,500,000 (US$65,217) per quality‐adjusted life year (QALY) gained was 99.5%.

Figure 3 shows the cost‐effectiveness acceptability curve (CEAC). With 1,000 ICERs produced by Monte Carlo simulations, the probability that the ICER was under the threshold was 99.5%.

The scenario sensitivity analysis assuming a fee of ¥1,600,000 for PBT yielded an ICER of ¥4,305,913/QALY gain, and the probability that the ICER was under the threshold by Monte Carlo simulation was 100.0%.

## 4 DISCUSSION

It has gradually become clear that PBT is clinically effective for LAPC and has fewer adverse events than conventional photon RTs, such as 3DCRT.^36^, ^37^, ^38^, ^39^, ^40^, ^41^ Compared to conventional photon RT, PBT is very attractive for the treatment of LAPC because it does not exacerbate effects on the gastrointestinal tract, even with dose escalation, and can be used concurrently with the same chemotherapy. The efficacy of photon RT using GEM and nab‐PTX for LAPC is currently being tested, and if promising results are obtained, a combination with PBT may be possible in future.^42^

At the same time, the higher costs of PBT in terms of equipment and power, compared to those of photon RT, raise concerns about its cost‐effectiveness and making it financially available to patients as another resource‐consuming healthcare innovation. In Japan, PBT for LAPC was recently covered under social insurance as an expansion of target cancers listed in the benefit package. To the best of our knowledge, Japan is the first country to make this addition. However, it has been processed without a formal cost‐effectiveness analysis commissioned by the authorities, as only a limited number (approximately 10) of innovations every year are subjected to obligatory economic appraisal in Japan's health system. PBT for LAPC was not selected by the authorities due to its smaller market size and lesser cost compared to that of the selected innovations.^35^ Till date, no report on its cost‐effectiveness has been published in academia or industry.

In this study, we examined the cost‐effectiveness of PBT for LAPC as a replacement for conventional RT and estimated its base‐case ICER value to be ¥ 5,376,915 (US$46,756)/QALY. This value is lower than ¥ 7,500,000 (US$65,217)/QALY gain, which is the willingness‐to‐pay (WTP) threshold for anti‐cancer treatment set by the authorities in Japan.^35^ PBT for LAPC was judged to be cost‐effective, and covering it under social insurance for patients is justifiable and an efficient use of extra healthcare resources. Thus, this replacement is socially acceptable in Japan from the perspective of health economics.

The one‐way sensitivity analysis found that the cost of PBT was the largest variability factor of the ICER range; however, the largest ICER was less than the threshold. Our results are considered stable against the uncertainty of the assumptions made in our model. The CEAC generated by the probabilistic sensitivity analysis showed that the probability of PBT being cost‐effective was 99.5%. This finding also supports the stability of our results.

A lower reimbursable fee for PBT might has been applied if LAPC was categorized as a non‐rare cancer. Using scenario sensitivity analysis for this case, the ICER was estimated to be ¥4,305,913/QALY. This value is not only lower than the threshold for anti‐cancer intervention, ¥7,500,000/QALY, but also a generic threshold for any innovation by the authority in Japan, ¥5,000,000/QALY. In this scenario, its cost‐effectiveness could be favorable against a stricter threshold, and the threshold for anti‐cancer therapy would not be necessary.

Although a number of cost‐effectiveness analyses of PBT have been reported worldwide,^43^, ^44^ this study is the first to report the cost‐effectiveness of PBT for LAPC. Transferring the results of cost‐effective analyses to other health systems is not straightforward, as both the assumed effectiveness and costs need to be scrutinized. Clinical evidence may differ due to practice or ethnicity, while costs depend on the financing system or economy. Nevertheless, our results suggest that replacing RT with PBT for LAPC could be cost‐effective in any health system. Making it available to patients as a standard treatment can be justified elsewhere.

This study has several limitations. First, the best clinical evidence underpinning better outcomes of PBT was based on a single‐arm and retrospective studies.^20^ There is also a wider range of eras included in 3DCRT reports than in PBT reports, which may have affected chemotherapy and supportive care after radiotherapy, which may have affected different outcomes.^26^ Second, adverse events were not considered. However, in published reports, adverse events of PBT, especially serious ones, were rare and more common in photon RT.^7^, ^23^, ^40^ Therefore, PBT is likely to be more cost‐effective in terms of dealing with adverse events. Third, the quality adjustment was not detailed. Differences among the chemotherapy regimens were not built in due to the availability of applicable utility weights in the literature. Fourth, there is no comparison with IMRT or SBRT. In Japan, national registries show that the number of cases of IMRT for LAPC is gradually increasing but many cases still use 3DCRT, so we compared 3DCRT and PBT in this study.^45^ Looking at the rate of IMRT use since 2015, there has been an increase until 2018, after which the rate has leveled off. On the contrary, the proportion of complex irradiation fields for 3DCRT continues to increase. In the future, as more patients are treated with IMRT for LAPC, cost‐effectiveness studies comparing IMRT with PBT will become more valuable. Lastly, this study focused on the cost‐effectiveness of PBT replacing photon RT in existing facilities and did not calculate the cost of building new facilities, where PBT for some other cancers has been included in the social insurance benefit package. Capital costs need to be considered in health systems where new facilities need to be built, while shared or overhead costs for PBT for other cancers need to be considered.

## 5 CONCLUSIONS

In conclusion, PBT is a cost‐effective replacement conventional photon RT, such as 3DCRT, for unresectable LAPC and such a replacement is socially acceptable in Japan from a health economics perspective.

## AUTHOR CONTRIBUTIONS

**Yuichi Hiroshima:** Conceptualization (lead); formal analysis (lead); methodology (lead); resources (lead); validation (lead); visualization (lead); writing – original draft (lead). **Masahide Kondo:** Conceptualization (lead); data curation (lead); investigation (lead); writing – review and editing (lead). **Takuya Sawada:** Data curation (equal); investigation (equal); resources (equal); validation (equal); writing – review and editing (equal). **Shu‐ling Hoshi:** Data curation (equal); investigation (equal). **Reiko Okubo:** Data curation (equal); investigation (equal). **Takashi Iizumi:** Writing – review and editing (equal). **Haruko Numajiri:** Writing – review and editing (equal). **Toshiyuki Okumura:** Supervision (lead). **Hideyuki Sakurai:** Project administration (lead).

## FUNDING INFORMATION

This work was partially supported by Grants‐in‐Aid for Scientific Research (B) (19H03596) from the Ministry of Education, Culture, Sports, Science, and Technology of Japan.

## CONFLICT OF INTEREST STATEMENT

The authors declare that they have no competing interests.

## ETHICS STATEMENT AND CONSENT TO PARTICIPATE

Not applicable.

## CONSENT FOR PUBLICATION

Not applicable.

## Data Availability

The datasets used and/or analyzed during the current study are available from the corresponding author on reasonable request.
